# First evidence of cholinesterase-like activity in Basidiomycota

**DOI:** 10.1371/journal.pone.0216077

**Published:** 2019-04-30

**Authors:** Kristina Sepčić, Jerica Sabotič, Robin A. Ohm, Damjana Drobne, Anita Jemec Kokalj

**Affiliations:** 1 Department of Biology, Biotechnical Faculty, University of Ljubljana, Ljubljana, Slovenia; 2 Department of Biotechnology, Jožef Stefan Institute, Ljubljana, Slovenia; 3 Department of Biology, Faculty of Science, Utrecht University, Padualaan, Utrecht, The Netherlands; Weizmann Institute of Science, ISRAEL

## Abstract

Cholinesterases (ChE), the enzymes whose primary function is the hydrolysis of choline esters, are widely expressed throughout the nature. Although they have already been found in plants and microorganisms, including ascomycete fungi, this study is the first report of ChE-like activity in fungi of the phylum Basidiomycota. This activity was detected in almost a quarter of the 45 tested aqueous fungal extracts. The ability of these extracts to hydrolyse acetylthiocholine was about ten times stronger than the hydrolytic activity towards butyrylthiocholine and propionylthiocholine. In-gel detection of ChE-like activity with acetylthiocholine indicated a great variability in the characteristics of these enzymes which are not characterized as vertebrate-like based on (i) differences in inhibition by excess substrate, (ii) susceptibility to different vertebrate acetylcholinesterase and butyrylcholinesterase inhibitors, and (iii) a lack of orthologs using phylogenetic analysis. Limited inhibition by single inhibitors and multiple activity bands using in-gel detection indicate the presence of several ChE-like enzymes in these aqueous extracts. We also observed inhibitory activity of the same aqueous mushroom extracts against insect acetylcholinesterase in 10 of the 45 samples tested; activity was independent of the presence of ChE-like activity in extracts. Both ChE-like activities with different substrates and the ability of extracts to inhibit insect acetylcholinesterase were not restricted to any fungal family but were rather present across all included Basidiomycota families. This study can serve as a platform for further research regarding ChE activity in mushrooms.

## Introduction

Cholinesterases (ChEs), the enzymes that hydrolyse choline esters but also exert non-hydrolytic activities [1], are considered as one of the catalytically most efficient enzymes in nature [2]. Cholinesterases are also widely expressed in organisms from different taxonomic groups [3]. It has been reported that ChEs with highly selective substrate specificity had appeared in the early bilaterians [4]. Two qualitatively different ChEs, acetylcholinesterase (AChE; E.C. 3.1.1.7) and butyrylcholinesterase (BChE; E.C. 3.1.1.8), were characterised in vertebrates. Phylogenetic analysis of vertebrate BChE and AChE expression indicate that these two enzymes have emerged from a common precursor whose function was to hydrolyse acetylcholine [5]. In some invertebrates (e.g. in crustaceans) it has been suggested that ChEs show intermediary characteristics between the two vertebrate forms and can be classified as neither AChE nor BChE [6,7]. ChEs have been found also in organisms devoid of nervous system such as sponges (Karczmar, 2010), both Gram positive and Gram negative bacteria [8–15], ascomycete fungi [16–18], plants [19–22], and protozoa [23–26]. However, no studies so far have reported the ChE-like activities in fungi belonging to the phylum Basidiomycota.

Most of the knowledge regarding the molecular structure of ChEs derives from studies on vertebrates. The first crystal structure of these enzymes was determined for AChE isolated from the electric organ of the Pacific electric ray (*Torpedo californica*) [27]. The active site of this enzyme has a conserved catalytic triad composed of amino acid residues Ser-Glu-His, and is located at the bottom of the deep and narrow active gorge located 2 nm from the enzyme surface. The active site generally comprises the anionic site, whose amino acid residues participate in cation-π interactions with the quaternary group of acetylcholine thus allowing its correct orientation; and the esteratic site, where the catalytic triad members participate in the hydrolysis of the substrate ester bond [27–29]. Another important site, the so-called peripheral anionic site, is located at the gorge entrance. Its aromatic amino acid residues participate in cation-π interactions with the quaternary group of acetylcholine [28–29], thus attracting the substrate and facilitating its way to the active site at the gorge bottom [30]. This site is also responsible for AChE inhibition by excess substrate [31]. The AChE-related enzyme, BChE, shares more than a 50% sequence homology with AChE, has a similar tertiary and quaternary structure, and also possesses a catalytic triad Ser-Glu-His located at the bottom of the gorge. There are however some differences between the two cholinesterases. As revealed from the crystal structure of human recombinant BChE [32], the structure of the active gorge of BChE allows for accommodation of larger substrates and wider substrate specificity. The peripheral anionic site of BChE is not inhibited by excess substrate [33], or by inhibitors that bind to the AChE peripheral site [34].

Several structurally distinct forms of ChEs have been reported in vertebrates and invertebrates and can be differentiated by the number and type of subunits [35–39]. In vertebrates, for example, at least eight forms of AChE were identified [3]. In invertebrates, ChEs are mostly globular [36, 39]. The molecular polymorphism of ChE appears to provide various possibilities of insertion of these enzymes in biological structures resulting in different physiological roles [40].

Vertebrate AChEs and BChEs have neuronal and non-neuronal functions as well as catalytic and non-catalytic activities [41]. The most commonly known physiological function of vertebrate AChE is in the termination of nerve impulses by hydrolysis of the neurotransmitter acetylcholine (ACh) in the cholinergic nervous system [3]. However, in vertebrates AChE is also involved in non-neuronal processes such as cell proliferation, differentiation, migration, ion and water movement, development and maintenance of synaptic and myoneural structures, and cell-cell communication [2, 42, 43] as well as its involvement in pathological states such as Alzheimer disease [44, 45]. Regarding BChE, several roles of this enzyme have been discovered in vertebrates, including modulation of cholinergic neurotransmission [46], hydrolysis of different toxic compounds, and participation in fat catabolism and an association with neuritic tissue degeneration [47].

The occurrence of cholinergic communication and regulation, and the presence of ChE enzymes early before neurogenesis, in anervous tissues, in (un)fertilized eggs and sperm, in unicellular organisms, and in fungi and plants implies non-neuronal functions of these enzymes [42]. While the proposed role of bacterial ChEs suggests their involvement in infection [8] or as natural toxins [11], it seems that in other organisms these enzymes play regulatory roles. As an example, ChEs were reported to be involved in the mating of *Paramecium primaurelia* [23], while plant ChEs were found to play a positive role in heat tolerance [21], in gravitropic response of the seedlings [22], and in water homeostasis and photosynthesis [48]. In invertebrates, reports suggest that ChEs play a role in fertilisation, embryogenesis [49, 50], tissue regeneration [51, 52], brood rearing [53], and xenobiotic defence [54, 55]. Although Pezzementi and Chatonnet [4] reported that the carboxylesterase family, with the subfamily cholinesterases, is broadly present in fungi, so far the non-neuronal functions of fungal ChEs have not been investigated and reported.

In this study, we investigated the ChE-like activities in fungi belonging to the phylum Basidiomycota. Mushrooms, the fruiting bodies of several basidomycota, have high value in medicine, the food industry and cosmetics [56–58]. The primary aim of this study was to characterise the ChE-like activity in mushrooms of 45 Basidiomycota species using different substrates and specific/unspecific vertebrate AChE and BChE inhibitors. We expected that, similar to invertebrate ChEs, across different Basidiomycote families there would be high variability in regards to the enzymes substrate specificities, the inhibitor specificities, and the ChE activity-substrate concentration relationship. We also expected that some aqueous fungal extracts will have the ability to inhibit insect AChE as previously shown for other fungal species [59–61]. Since some fungal lipases show similarity to vertebrate AChE [62, 63], we checked whether these aqueous mushroom extracts show lipase activity and if this activity correlates to the ability to hydrolyse choline esters. This basic study on the presence of ChE-like activity in mushrooms may provide a basis for further research on potential non-neuronal roles of these enzymes in fungi. Moreover, it could contribute to our understanding of the various functions of these remarkable enzymes which, until recently, were thought to be limited mostly to vertebrate neurons.

## Materials and methods

### Chemicals

Bideionized water (MQ) with a resistivity of 18.2 MΩ⋅cm (Milli-Q Advantage A10, MILLIPORE) was used for standards and sample solution preparation. Sodium hydrogencarbonate (NaHCO_3_); 5,5′ dithiobis-2-nitrobenzoic acid (DTNB); propionylthiocholine chloride (PCh); CuSO_4_, trisodium citrate, *p*-nitrophenyl butyrate and bovine serum albumine (BSA) were all of analytical grade and purchased from Sigma-Aldrich (USA). Acetylthiocholine chloride (ACh) was form Sigma-Aldrich (UK) and butyrylthiocholine chloride (BCh) from Sigma-Aldrich (Switzerland). BCA Protein Assay Reagent A and BCA Protein Assay Reagent B were purchased from Pierce (Thermo Fisher Scientific, USA). Tris and glycine were from Serva (Germany); HCl, acetic acid, and glycerol from Carlo Erba (Italy); acetate and bromphenol blue from Kemika (Croatia); and potassium ferricyanide from Fluka (Switzerland). The 96-well flat base, transparent, polystyrene plates were used for enzyme assays (Sarstedt, Germany). All buffer solutions were filter-sterilized through a 0.22 μm filter (Millipore, USA). The following ChE inhibitors were used: trichlorfon or metrifonate (Sigma-Aldrich, Germany), Iso-OMPA (tetraisopropyl pyrophosphoramide) (Sigma-Aldrich, UK), neostigmine bromide (Sigma-Aldrich, Japan), edrophonium chloride (Sigma-Aldrich, Germany), BW284C51 (Sigma-Aldrich, Israel) and ethopropazine chloride (Sigma-Aldrich, Germany). *Drosophila melanogaster* AChE was a kind gift of Professor Jean-Louis Marty (Perpignan, France), while electric eel (*Electrophorus electricus*) AChE was from Sigma-Aldrich (USA).

### Selected mushroom species

Mushrooms (phylum Basidiomycota) were collected from grasslands and forest stands at various locations in western and central Slovenia, identified to species level and stored at -20°C. Taxonomic classification follows the Index Fungorum database (http://www.indexfungorum.org). Mushrooms collected for analysis were assigned numerical IDs in a random order, but not all were included in this study. However, to preserve tracking of the obtained data the initial codes were kept to ID the mushrooms (Table 1).

**Table 1:** A summary of all characteristics for 45 aqueous mushroom extracts which are listed according to their taxonomic position.

Order	Family	Species	ID	Edibility^a^	Growth type^b^	BCh hydrolysing activity			PCh hydrolysing activity			ACh hydrolysing activity			Lipase	*Drosophila* AChE inhibition
						1^c^	3	5	1	3	5	1	3	5		
Agaricales	Strophariaceae	*Cyclocybe aegerita*	49	e	s											
	Hymenogastraceae	*Hypholoma fasciculare*	9	p	s											
		*Gymnopilus penetrans*	24	i	s											
	Cortinariaceae	*Cortinarius purpurascens*	17	e	m											
		*Cortinarius variicolor*	18	e*	m											
		*Cortinarius violaceus*	6	e	m											
	Psathyrellaceae	*Coprinopsis atramentaria*	36	e*	s											
	Agaricaceae	*Echinoderma asperum*	13	p	s											
		*Echinoderma echinaceum*	32	i	s											
		*Lycoperdon perlatum*	30	e*	s											
		*Lycoperdon pyriforme*	4	e*	s											
		*Lepiota brunneoincarnata*	15	p	m											
		*Macrolepiota procera*	21	e	s											
	Entolomataceae	*Entoloma rhodopolium*	1	p	m											
	Tricholomataceae	*Clitocybe gibba*	35	e	s											
		*Clitocybe nebularis*	42	e*	s											
		*Clitocybe phaeophthalma*	31	p	s											
		*Infundibulicybe geotropa*	40	e	s											
		*Tricholoma pardinum*	12	p	m											
		*Tricholoma sejunctum*	8	e*	m											
		*Tricholoma sulphureum*	3	p	m											
		*Tricholomopsis rutilans*	14	i	s											
	Physalacriaceae	*Armillaria borealis*	16	e*	s											
		*Armillaria ostoyae*	33	e*	s											
		*Mucidula mucida*	11	e	s											
	Pleurotaceae	*Pleurotus ostreatus*	51	e	s											
	Amanitaceae	*Amanita citrina*	7	e	m											
		*Amanita excelsa*	29	e	m											
		*Amanita phalloides*	2	p	m											
		*Amanita rubescens*	10	e*	m											
	Hygrophoraceae	*Hygrophorus eburneus*	38	e	m											
Boletales	Hygrophoropsidaceae	*Hygrophoropsis aurantiaca*	37	e*	s											
	Gomphidiaceae	*Gomphidius glutinosus*	19	e	m											
	Suillaceae	*Suillus variegatus*	5	e	m											
	Sclerodermataceae	*Scleroderma citrinum*	20	p	m											
	Boletaceae	*Caloboletus calopus*	28	i	m											
		*Xerocomellus chrysentheron*	27	e	m											
Russulales	Russulaceae	*Lactarius rubrocinctus*	41	i	m											
		*Lactarius torminosus*	39	e*	m											
		*Russula ochroleuca*	34	e*	m											
		*Russula torulosa*	43	p	m											
	Bondarzewiaceae	*Heterobasidion sp*.	50	i	s											
Polyporales	Polyporaceae	*Trametes versicolor*	48	i	s											
Geastrales	Geastraceae	*Geastrum fimbriatum*	25	i	s											
Cantharellales	Cantharellaceae	*Cantharellus cinereus*	22	e	m											

The ChE-like activity was ranked in four groups depending on the activity values (nmol of hydrolysed substrate/ mg protein. min): blue (0), yellow (0–0.1); orange (0.1–0.5); red (> 0.5). For each substrate (ACh, PCh and BCh) three concentrations were shown: 1, 3, and 5 mM^c^. Lipase activity was ranked in four groups depending on the activity values (ng *p*-NP/mg protein): blue (0), yellow (0–10); orange (10–25); red (> 25). Aqueous extracts with inhibitory potential against *Drosophila* AChE are shown as dark blue and those without this ability as blue. All mushrooms belong to phylum Basidiomycota, Agaricomycotina, Agaricomycetes

^a^*Edibility*: e, edible; e* conditionally edible; i, inedible; p, poisonous

^b^*Growth type*: s, saprotrophic; m, mycorrhizal

### Phylogenetic analysis of putative ChE genes in the fungal kingdom

Predicted proteins of 12 basidiomycetes, 5 ascomycetes and 3 early diverging fungi (S1 Table) were used in a phylogenetic analysis of putative ChE genes. Known animal ChE proteins were used as a reference (S2 Table), as well as the predicted proteins from the human genome (Build 38 patch 11). Homologs of known ChE proteins were identified by the presence of a PF00135 PFAM domain [64], which represents a carboxylesterase domain. The identified domains are aligned using MAFFT version 7.307 [65] using local pairwise alignment and 1000 iterations. FastTree 2.1 was used to calculate a gene tree with default settings [66]. iTol was used for tree visualization and curation [67]. Manual curation of previously published predicted proteins was beyond the scope of this study.

### Preparation of aqueous extracts

Frozen mushroom specimens (5 g) were homogenized in 10 mL of 100 mM Tris-HCl buffer, pH 7.4, and extracted for 12 hours with constant shaking (400 rpm at 4°C) followed by centrifugation (11900×*g* at 4°C). Supernatants were removed and stored in aliquots of 1 mL at -20°C.

### Colorimetric ChE activity assay

ACh, PCh and BCh-hydrolysing activities were measured according to the Ellman’s method [68] with minor modifications, using a VIS microplate reader (Infinite F Nano^+^; Tecan, Switzerland). The initial reaction mixture per well was: 50 μL of aqueous extracts and 100 μL of Ellman’s reagent which was prepared by dissolving 91 mg of DTNB and 37.5 mg of NaHCO_3_ in 1 L of potassium phosphate buffer (KPB) (100 mM, pH 7.4). Prior to addition of the substrates endogenous reactions with only the aqueous extracts and Ellman's reagent were run at 25°C for 15 min and the absorbances of these reaction were assessed at 405 nm. These accounted for the reactions of intrinsic thiol groups with DTNB. After this period, the substrates (50 μL) were added to start the enzymatic reactions at 25°C for 15 min, which were followed at 405 nm. Finally, the endogenous reaction slopes were subtracted from the slopes obtained after the addition of the substrates.

Activities were measured in final concentrations of 1, 3 and 5 mM. The activities of all aqueous extracts were measured in triplicates. ChE-like activity was expressed in nmoles of hydrolysed substrates min^-1^ mg^-1^ of the proteins (extinction coefficient, ε_405_ = 13,600 M^−1^ cm^−1^). The concentration of proteins in aqueous extracts was measured using a modification of the bicinchoninic acid protein assay per the manufacturer’s manual (Pierce, Rockford, IL, USA) with BSA as a standard. The absorbance was determined at 550 nm using a microplate reader (Dynex Technologies, USA) after 30 min of incubation in the dark at 37°C. All aqueous extracts were measured in quadruplicates.

### In-gel detection of ChE-like activity

Cholinesterase-like activity using ACh as a substrate was analysed using polyacrylamide gel electrophoresis under non-denaturing conditions and staining [69]. Mushroom aqueous extracts (30 μl) were mixed with buffer (0.125 M Tris-HCl, pH 6.8, 20% glycerol, 0.5% Triton-X-100 with bromphenol blue), incubated at room temperature for 10 min and electrophoresed in 8% polyacrylamide resolving gel containing 0.5% Triton-X-100 in Tris/glycine buffer (pH 8.3) with 0.5% Triton-X-100 at constant voltage 120 V for 70 min while cooled in an ice bath. Gels were washed in dH_2_O for 10 min and then incubated in developing buffer (67 mM phosphate buffer or 100 mM maleate buffer, pH 6, with 5.5 mM citrate, 3.3 mM CuSO_4_, 0.5 mM potassium ferricyanide and 5 mM ACh) at room temperature from 2 to 24 h. Gels were documented using an image scanner. Alternatively, acid pH non-denaturing conditions were used for resolving gel (0.5 M acetic acid, pH 4, with 0.5% Triton-X-100), buffer (50% glycerol) and running buffer (40 mM β-alanin with acetic acid, pH 4, and 0.5% Triton-X-100).

To determine the molecular mass of selected fungal ChE-like enzymes, the activity bands were excised from the gel run under non-denaturing conditions after 2 to 4 h incubation in developing buffer. Proteins were eluted from the gel pieces by incubation in SDS loading buffer overnight at 4°C followed by boiling for 10 min and then analysed in 10% SDS-PAGE by silver staining. Low Molecular Weight markers of 14.4–97 kDa (GE Healthcare) were used for molecular mass estimation.

### Inhibition of ChE-like activities

After the initial screening of 45 mushroom aqueous extracts, 8 extracts that showed evident activity with ACh (Table 1) were further used to investigate the inhibition by known vertebrate AChE and BChE inhibitors. The following inhibitors were tested: trichlorfon or metrifonate, Iso-OMPA (tetraisopropyl pyrophosphoramide), neostigmine bromide, edrophonium chloride, ethoproprazine chloride and BW284C51. Stock solutions of inhibitors were prepared in dH_2_O. The initial reaction mixture per well was: 10 μL of inhibitor (final concentration in the well; 1 mM), 50 μL of extract and 100 μL of Ellman’s reagent. Initially, a 15-min endogenous reaction was run as described in section 2.5. Afterwards, 50 μL of ACh (final concentration 3 mM) was added to start the ChE reaction (followed at 405 nm and 25°C for 15 min). All necessary blanks with buffer (100 mM, pH 7.4) instead of inhibitor were run. All the reactions were measured in triplicates.

### Lipase activity assay

The lipase activity of aqueous extracts was assayed quantitatively using a spectrophotometric method with *p*-nitrophenyl butyrate (*p*-NPB) as a substrate, as described [70]. The measurements were performed in triplicates. Aqueous extracts were replaced by extraction buffer in negative control measurements. Briefly, 450 μL of buffer (100 mM sodium phosphate buffer supplemented with 150 mM NaCl and 0.5% (v/v) Triton-X-100, pH 7.2) and 5 μL of 50 mM *p-*NPB (dissolved in acetonitrile) were mixed in Eppendorf tubes and incubated for 10 min at 37°C. Following incubation, 45 μl of aqueous extracts were added to the mixtures and incubated for an additional 10 min at 37°C. The reactions were stopped by adding 750 μl of acetone. All the reaction mixtures were centrifuged for 10 min at 5000×*g* and 22°C. Following centrifugation, 200 μl of the supernatants were transferred to the wells of a 96-well microplate where the production of *p*-nitrophenol (*p-*NP) was monitored at 405 nm using a microplate reader (Dynex technologies, USA). Lipase activity was defined as the release of *p-*NP (in μmol) in 1 min per 1 mg of soluble proteins. All the reactions were measured in triplicates.

### Inhibition of insect AChE activities by mushroom aqueous extracts

We investigated the potential of these 45 aqueous extracts to inhibit *D*. *melanogaster* AChE. Insect AChE was chosen because there is a reasonable likelihood that insects and mushrooms will interact in their natural habitat. The AChE activity was measured according to Ellman’s method [68] analogous to section 2.5. The reaction mixture per well was: 100 μL of Ellman’s reagent, 20 μL of aqueous extract, 50 μL of ACh (final concentration 0.5 mM) and 50 μL of insect AChE (0.4 U/mL). The reaction was followed at 405 nm and 25°C for 5 min. All the reactions were measured in triplicates.

## Results

### ChE-like activity in mushroom aqueous extracts using different substrates

**Fig 1:**
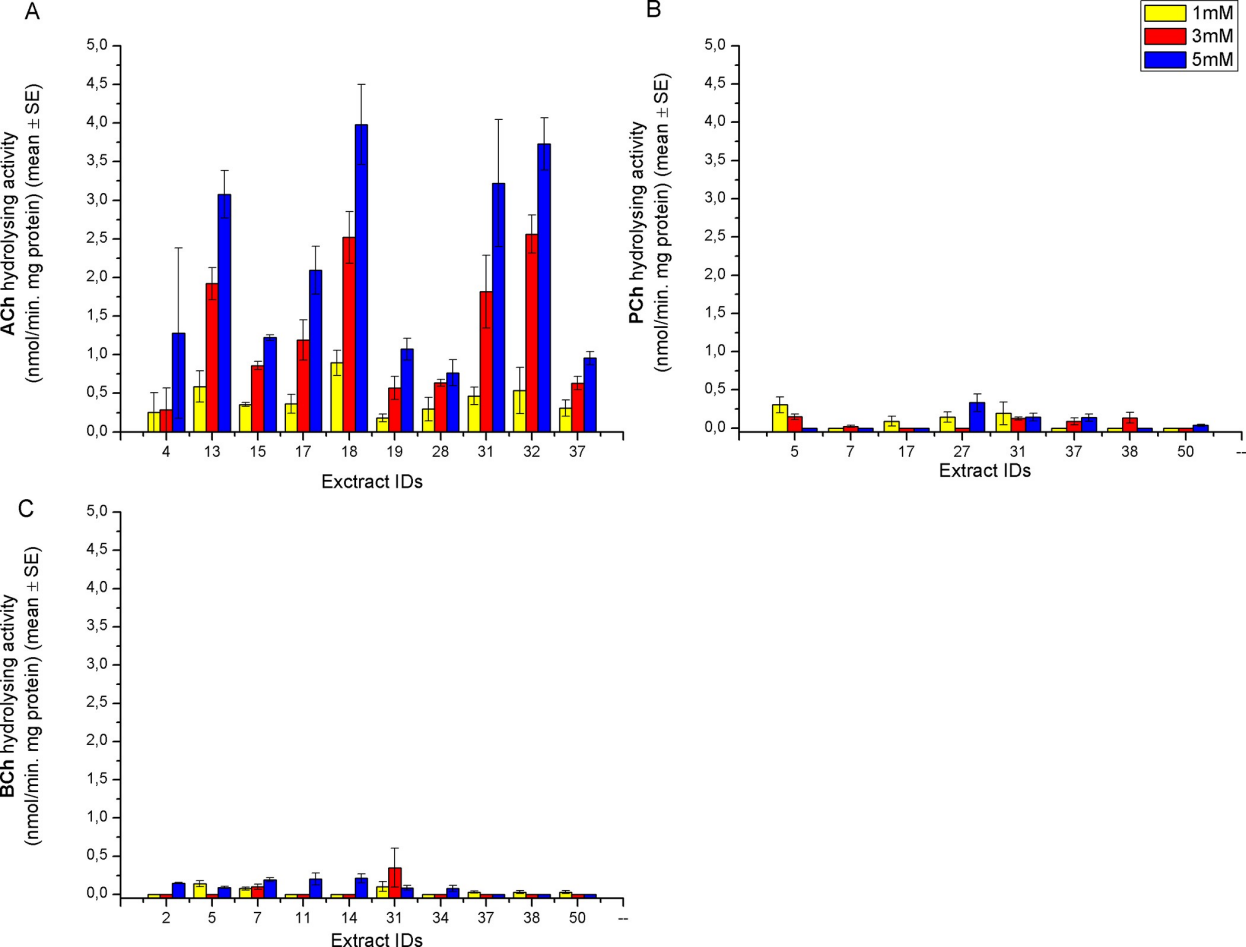
**Cholinesterase-like activity of aqueous extracts of analysed Basidiomycota using acetylthiocholine chloride (ACh) (A), propionylthiocholine chloride (PCh) (B) and butyrylthiocholine chloride (BCh) (C) as substrates.** The activity of each extract is shown in respect to the concentration of the substrate (final conc. 1, 3, and 5 mM). Only the extracts with apparent enzyme activity are shown. The rest of the extracts had no recorded ACh/ BCh/ PCh hydrolysing activity. Please note that the list of active aqueous extracts differs with different types of substrates. Mean values of four independent biological repetitions with corresponding standard errors are shown. Aqueous extracts IDs denote: 2-*Amanita phalloides;* 4-*Lycoperdon pyriforme;* 5-*Suillus variegatus;* 7- *Amanita citrina; 11-Mucidula mucida; 13-Echinoderma asperum; 14-Tricholomopsis rutilans; 15-Lepiota brunneoincarnata; 17-Cortinarius purpurascens; 18-Cortinarius variicolor; 19-Gomphidius glutinosus;* 27-*Xerocomellus chrysentheron; 28-Caloboletus calopus;* 31-*Clitocybe phaeophthalma*; 32-*Echinoderma echinaceum*; *34-Russula ochroleuca;* 37-*Hygrophoropsis aurantiaca*; *38-Hygrophorus eburneus; 50-Heterobasidion sp*.

All 45 mushroom aqueous extracts were assayed for ChE-like activity using ACh, PCh and BCh as substrates. Ten extracts out of 45 showed activity with ACh, 8 with PCh and 10 with BCh. Six extracts that hydrolysed BCh (IDs: 5, 7, 31, 37, 38, 50) were also active using PCh. In contrast, only two extracts that hydrolysed ACh (IDs: 31 and 37) were also active with BCh and three extracts (IDs: 17, 31, 37) also showed PChE-like activity (Fig 1).

Absolute ChE-like activities of mushroom aqueous extracts were the highest in the case of ACh, while those of BCh and PCh were approximately an order of magnitude lower at respective substrate concentrations. The ChE-like activity increased with increasing concentration of ACh (from 1 to 5 mM). In the case of BCh and PCh, no general rule regarding the correlation between the concentration of the substrates and the activity could be deduced (Fig 1).

### In-gel detection of ChE-like activity in mushroom aqueous extracts

**Fig 2:**
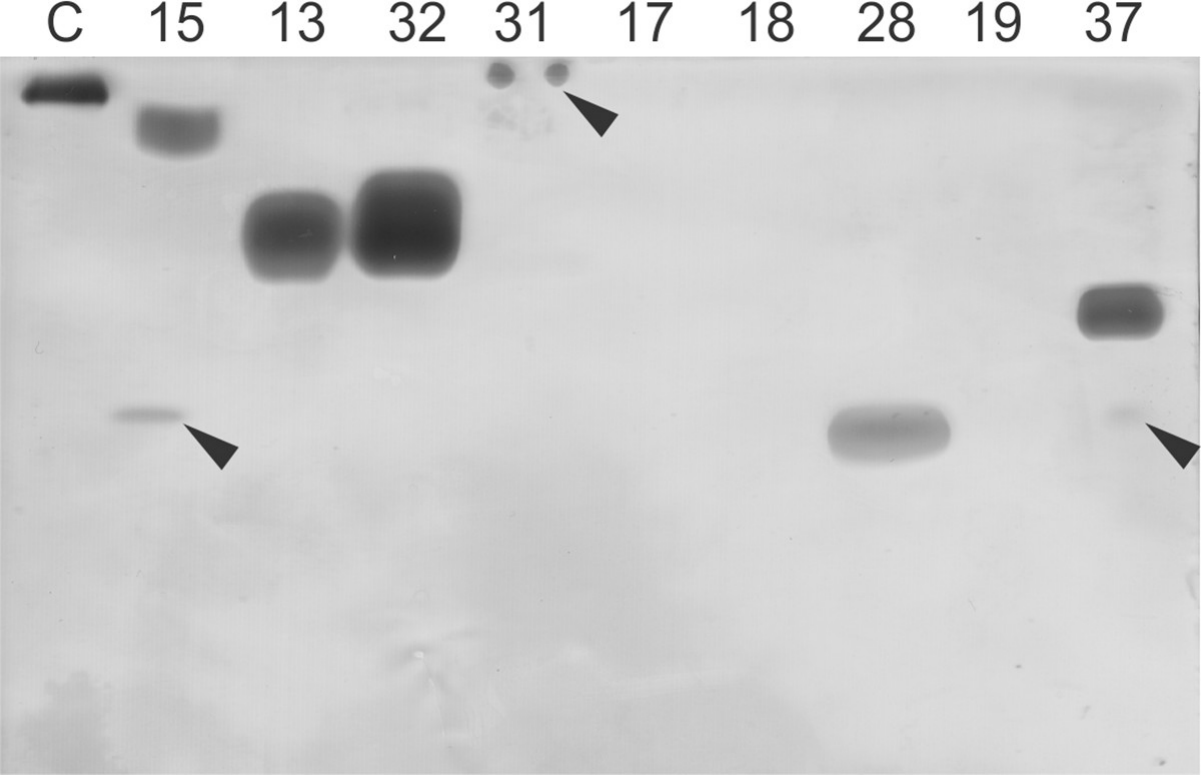
In-gel cholinesterase-like activity of aqueous extracts of selected species of Basidiomycota. Gels were developed in phosphate buffer at pH 6 with 5 mM acetylthiocholine chloride overnight at room temperature. Electric eel acetylcholinesterase was used as control (C). Weak activity bands are marked with black arrows. IDs represent the following species: 15-*Lepiota brunneoincarnata*; 13-*Echinoderma asperum*; 32-*Echinoderma echinaceum*, 31-*Clitocybe phaeophthalma*; 17-*Cortinarius purpurascens*; 18-*Cortinarius variicolor*; 28-*Caloboletus calopus*; 19-*Gomphidius glutinosus*; 37-*Hygrophoropsis aurantiaca*.

In-gel detection of ChE-like activity with ACh revealed an unexpected diversity of ChE-like enzymes in mushrooms (Fig 2) that suggested the existence of different molecular forms (e.g. different molecular masses, isoelectric points and/or oligomeric forms). Comparison of the relative mobility of fungal ChE-like activity bands to that of the electric eel AChE, with the pI at pH 5.3 and molecular mass of 280 kDa composed of four 70 kDa-subunits, suggests that fungal enzymes have higher isoelectric points and/or lower molecular masses. Higher pI of mushroom ChE-like enzymes was also corroborated by their lack in mobility in the native polyacrylamide gel run at acidic pH (S1 Fig). Furthermore, analysis of the activity bands of the species *Echinoderma echinaceum* (ID 32) and *Hygrophoropsis aurantiaca* (ID 37) showed a molecular mass of the putative monomeric subunits of 60 and 65 kDa, respectively. Some additional lower molecular mass bands were noted that could represent proteins associated with the active ChE in a protein complex, or its degradation products due to proteases present in the crude extracts (S2 Fig).

The in-gel determined hydrolysing activity with ACh (Fig 2) was not confirmed for all aqueous extracts that showed the activity with this substrate in the colorimetric assay (Fig 1A). No in-gel detected ChE-like activity was found for aqueous extracts 17, 18 and 19, and only weak activity was shown for aqueous extract 31. The samples that showed very high activity in the colorimetric assay did not necessary show high activity in-gel (e.g. IDs 18 and 31) and *vice versa* (e.g. IDs 37), as determined by the intensity of the band. These discrepancies could be the consequence of different limits of detection in both assays or can derive from the fact that some ChE-like enzymes are not stable enough to withstand the in-gel detection procedure. Furthermore, some enzymes can be bound by inhibitors that mask the activity detection in solution but not in gel, where they can be separated by the electrophoresis.

### Lipase activity in mushroom aqueous extracts

**Fig 3:**
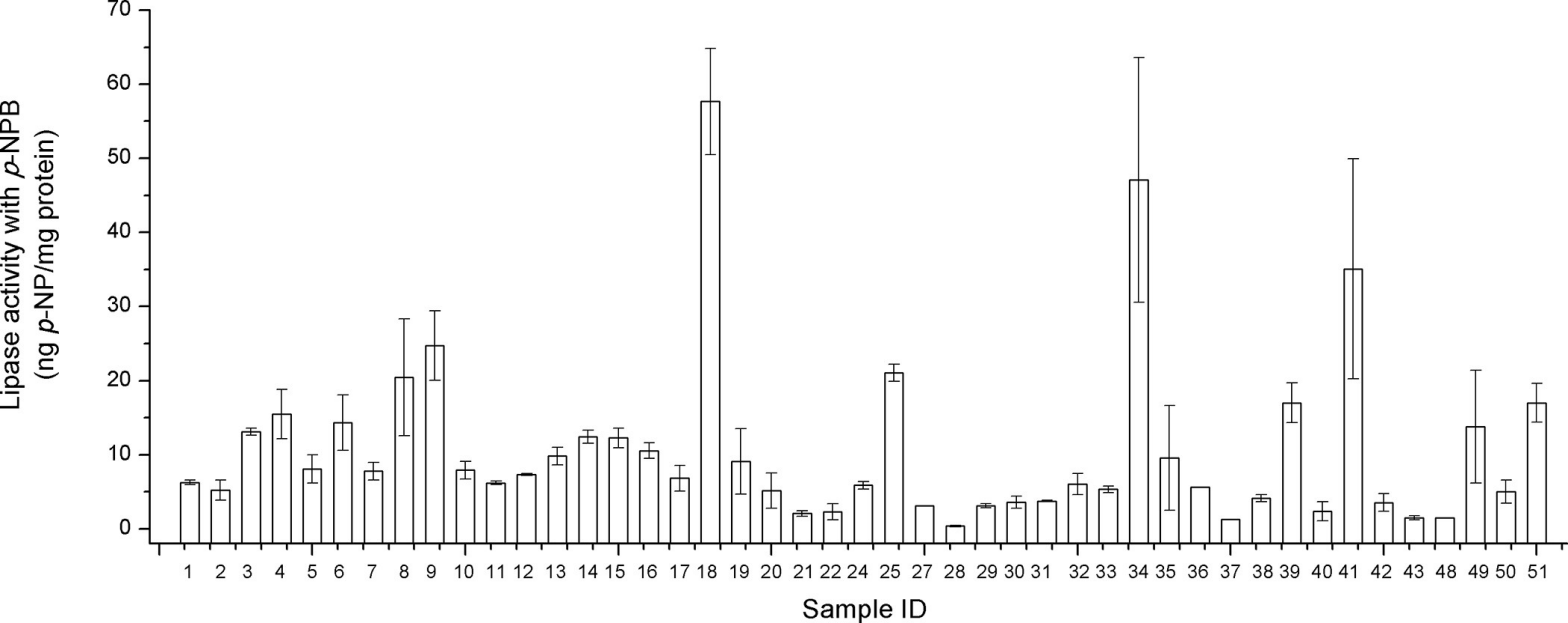
Lipase activity in mushroom aqueous extracts. Mean values of three independent biological repetitions with corresponding standard errors are shown. Please refer to Table 1 for the list of mushroom species. *p*-nitrophenyl butyrate (*p*-NPB) was used as a substrate.

Lipase activity was detected in almost all analysed aqueous mushroom extracts. The majority of these (61%) exerted the lipase activity in the range of up to 10 ng *p*-NP/mg protein, 33% in the range of 10–25 ng *p*-NP/mg protein, while three aqueous extracts (IDs 18, 34 and 41, corresponding to species *Cortinarius variicolor*, *Russula ochroleuca* and *Lactarius rubrocinctus* had evidently higher activity (>25 ng *p*-NP/mg protein) (Fig 3).

### Influence of different inhibitors on ChE-like activity in mushroom aqueous extracts

**Fig 4:**
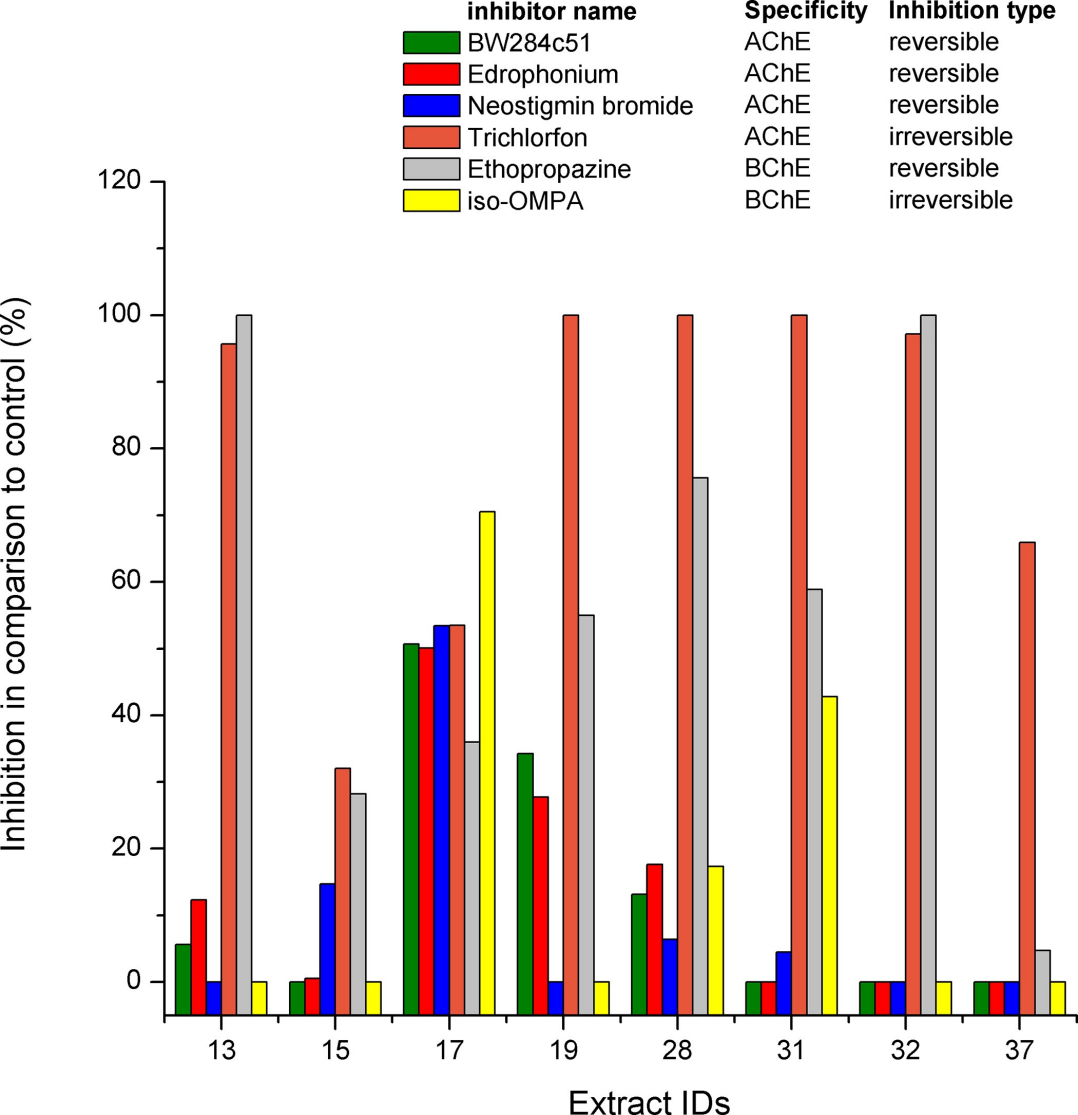
Inhibition of cholinesterase-like activity with acetylthiocholine chloride (ACh) as a substrate by selected inhibitors (final concentration of each inhibitor was 1 mM). Only selected aqueous extracts showing activity with ACh (Fig 1A) were tested. Inhibitors are listed according to their type of inhibition (reversible, irreversible) and their distinctive inhibitory affinity against vertebrate AChE or BChE. Mean values of three independent biological repetitions with corresponding standard errors are shown. IDs represent the following species: 13-*Echinoderma asperum*, 15-*Lepiota brunneoincarnata*; 17-*Cortinarius purpurascens*; 19-*Gomphidius glutinosus*; 28-*Caloboletus calopus;* 31-*Clitocybe phaeophthalma*; 32-*Echinoderma echinaceum*; 37-*Hygrophoropsis aurantiaca*.

We tested whether established inhibitors for vertebrate AChE and BChE would inhibit the activity of fungal ChE-like enzymes using ACh as a substrate. Only the selected aqueous extracts showing evident activities with ACh were assayed (Fig 4). Reversible AChE inhibitors (BW284c51, edrophonium chloride and neostigmine bromide) had either no (IDs 15, 31, 32, 37) or very little (up to 20–30%) inhibitory effect (IDs 13, 15, 19, 23, 28) on the hydrolysing activity with ACh. Trichlorfon, an irreversible vertebrate AChE-specific inhibitor, showed the highest inhibitory effect in all aqueous extracts. Almost complete inhibition (100%) was recorded for five extracts (IDs: 13, 19, 28, 31, 32), extract ID 37 was inhibited by 70%, extract ID 17 by 50%, and extract ID 15 by 30%. The *Iso*-OMPA, an irreversible vertebrate BChE specific inhibitor, had no effect on 67% of the samples. Extracts IDs 28, 31 and 17 were inhibited by this inhibitor by 20%, 45%, and 70%, respectively. All inhibitors had the lowest impact on extract ID 15 (*Lepiota brunneoincarnata*).

### Inhibitory activity of some mushroom aqeous extracts against insect AChE

**Fig 5:**
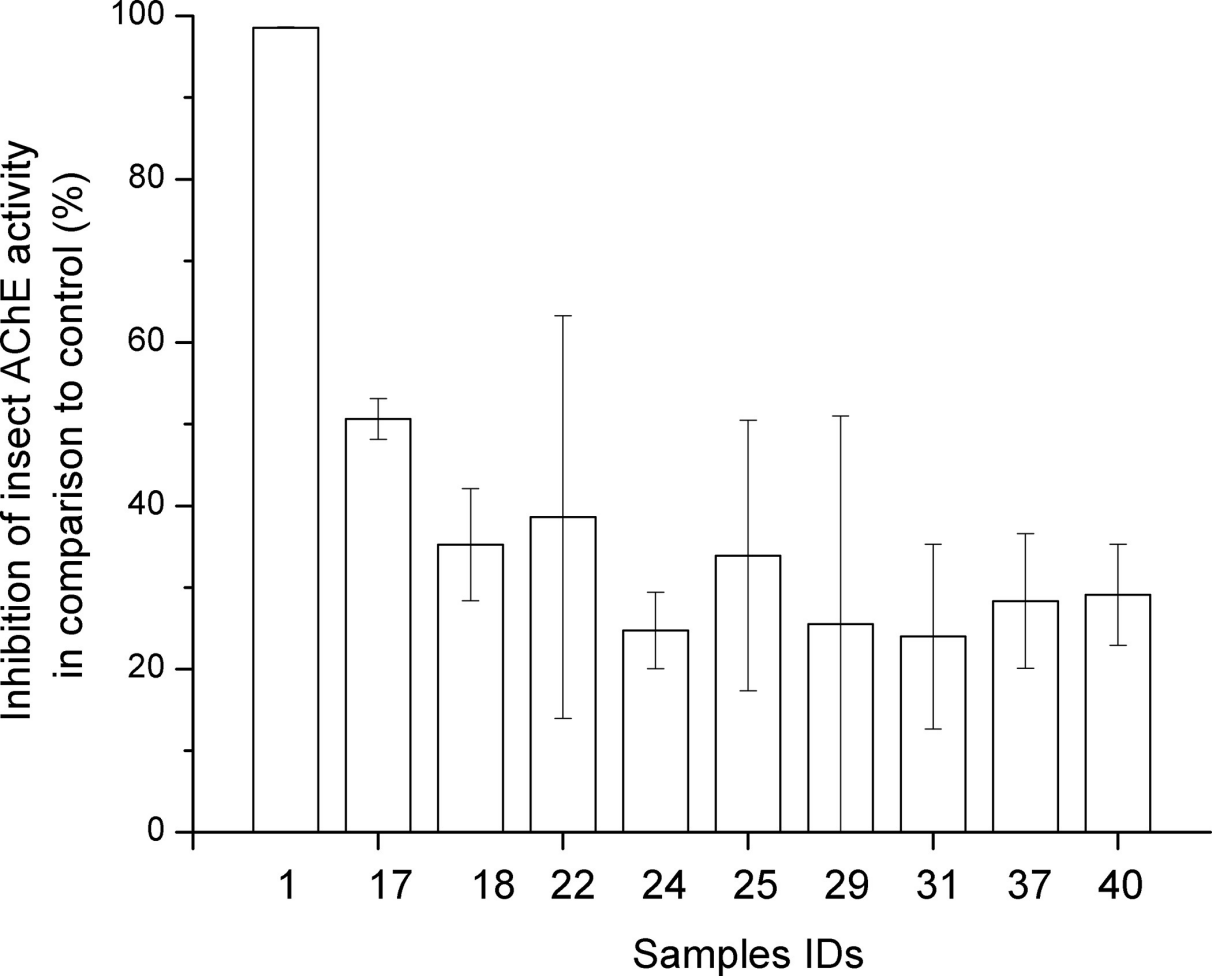
Inhibition of *Drosophila melanogaster* acetylcholinesterase by active mushroom extracts. The rest of the extracts showed no evident inhibition. Mean values of three independent biological repetitions with corresponding standard errors are shown. IDs represent the following species: 1-* Entoloma rhodopolium;* 17-*Cortinarius purpurascens*; 18-*Cortinarius variicolor¸* 22-* Cantharellus cinereus;* 24-*Gymnopilus penetrans;* 25-*Geastrum fimbriatum;* 29-*Amanita excelsa;* 31-*Clitocybe phaeophthalma;* 37-*Hygrophoropsis aurantiaca*; 40- *Infundibulicybe geotropa*.

Our data show that only 22% of the mushroom aqueous extracts (IDs 1, 17, 18, 22, 24, 25, 29, 31, 37, and 40) possess compounds that act as inhibitors of *D*. *melanogaster* AChE (Fig 5). Most of these extracts inhibited insect AChE activity in the range of 20–40% in comparison to the untreated enzyme, while only extract 1 had a 100% inhibitory potential. This indicates that *E*. *rhodopolium* extract contains compound(s) that act as strong and specific inhibitor(s) of *Drosophila* AChE.

### Phylogenetic analysis of putative ChE proteins in the fungal kingdom

Known ChE proteins from animals have a conserved carboxylesterase domain (Pfam domain PF00135), which can be used to find homologs in the fungal kingdom. However, this domain is not unique to ChE proteins and is also found, for example, in lipases [64]. First, we used the conserved domain to identify homologs of ChE proteins in the genomes of 22 members of the fungal kingdom (S1 Table). There is a wide variation in the number of homologs, ranging from none in *S*. *cerevisiae* to 37 in *F*. *graminearum*. Next, we calculated a gene tree of these fungal homologs in order to discriminate between (putative) true ChE proteins and more general carboxylesterases (including lipases). As a reference for ChE proteins we included known animal ChE proteins, and as a negative reference we included all other human proteins with Pfam domain PF00135 (S3 Fig). The gene tree reveals that there are no clear orthologs of ChE enzymes in fungi, since the known ChE proteins (in red) are closer related to human non-ChE homologs (in blue, including lipases) than to any fungal homolog (in black). This suggests that in animals the carboxylesterase family of proteins diversified into its various functions (including ChE) after the split between animals and fungi.

## Discussion

To the best of our knowledge, higher fungi have not been a matter of ChE activity investigations up to now, and this study provides the first evidence of ChE-like activity in Basidiomycota using ACh, PCh and BCh as substrates. ChE activity was reported in several species of the phylum Ascomycota (genera *Aspergillus*, *Penicillium* and *Fusarium*) in the early '90s [16–18]. However, the genes encoding these enzymes have not been identified. Interestingly, acetylcholine, a naturally occurring ChE substrate, has been detected in the extract of the ergot fungus, *Claviceps purpurea*, more than a hundred years ago [71].

An established mode of ChE activity characterisation is to investigate their substrate specificities, ChE activity-substrate concentration relationship and inhibitor specificities. Typically, vertebrate AChE is distinguished from vertebrate BChE by the following criteria: (i) AChE is typically inhibited by 10^−5^ M BW284c51 whereas BChE by 10^−5^ M iso-OMPA; (ii) AChE hydrolyses ACh faster than PCh and does not cleave BCh, which is the optimal substrate for BChE; and (iii) only AChE presents excess (10^−3^ M ACh) substrate inhibition. Cholinesterases are distinguished from nonspecific esterases, which do not use choline esters as optimal substrates, by their sensitivity to 10^−5^ M eserine sulphate (physiostigmine) [72]. Below, we discuss each of these properties in regard to the ChE-like activity of aqueous mushroom extracts tested in this study.

Approximately twenty percent of all aqueous extracts showed evident hydrolysing activity with ACh, PCh and BCh, however, these extracts do not belong to the same mushroom species. Only 20% and 30% of aqueous extracts that hydrolysed ACh also hydrolysed BCh and PCh, respectively. Cholinesterase-like activities were the highest in the case of ACh (up to 5 nmol/min. mg protein), while those of BCh and PCh were 10 to 50-times lower. In the case of some invertebrates, ChE typically cleaves ACh and PCh with similar preferences, but the activity using BCh is commonly 10-times lower [6,7, 39, 73–75]. There are also exceptions, seen in some crustacea, where ChE preferentially cleaves BCh [39]. Bacterial, plant and protozoan ChEs hydrolyse ACh and PCh but none have shown the ability to cleave BCh [8, 9, 12, 13, 14, 19, 21–24]. This data indicates great variability in substrate affinities among ChEs from different organisms, which was also observed for our mushroom extracts. The ability of mushroom extracts to hydrolyse ACh, BCh and/or PCh seems to be a species- and not family-specific trait (Table 1). This excludes a truly essential physiological role of a cholinergic system in this phylum.

We suggest that in further studies more attention should be paid also to investigate the ChE-like activities in different parts of mushrooms, since the current study was done on composite samples. Namely, it has been reported that ACh content varies depending on the part of the *Lentinus edodes* mushroom, being more than 2-fold higher in the stem that in the cap [76]. This may suggest that the ChE-like activity also differs in distinct mushroom parts, although the presence of ACh does not explicitly imply the presence of ChEs Furthermore, developmental stage, growth site characteristics and environmental factors may all affect the expression of ChE-like activity, as has been shown for other enzymes and proteins in mushrooms [57, 77]. In our study, the absence or presence of ChE-like activity in certain fungal species was not related to their lifestyle, their mycorrhizal or saprotrophic status for example (Table 1). It could be possible that these enzymes have a role in resistance to predators or grazing, since there are previous reports of ChEs acting as toxins (Nieto et al., 1991). However, the physiological role of these choline esters—hydrolysing enzymes needs to be explored further.

ChE-like activities in mushroom extracts were not inhibited at high substrate concentrations (up to 5 mM). A similar pattern was found for plant ChEs [19, 20], but most of bacterial ChEs [12– 14] and invertebrate ChEs [6, 75] showed inhibition at high substrate concentrations. Vertebrate AChEs are characterised by high substrate inhibition, so despite preferential ACh hydrolysis (**Fig 1**), mushroom ChE-like enzymes- in our study cannot be classified as vertebrate-like AChEs.

None of the mushroom extracts was inhibited by known reversible vertebrate AChE inhibitors (BW284c51, edrophonium chloride and neostigmine bromide which is considered an analogue of eserine) [72] which may denote that mushroom enzymes bearing ChE-like activity may actually be non-specific hydrolases. This has also been questioned in the case of invertebrates [75]. Contrary to our data, it has been shown that ChE from the filamentous fungus *Aspergillus niger* was inhibited by neostigmine and eserine sulphate [18]. However, trichlorfon, the irreversible vertebrate AChE specific inhibitor, induced the highest inhibitory effect to all mushroom aqueous extracts with almost complete inhibition in 5 out of 8 extracts implying that these enzymes have an active site with the active serine that covalently binds organophosphates. The irreversible vertebrate BChE-specific inhibitor, *iso*-OMPA, only inhibited three out of eight extracts which were inhibited by 20–70%. Only one of these three showed a preference to BCh, therefore, we cannot classify these aqueous extracts as vertebrate BChE-like. Similar differences in sensitivity to AChE and BChE inhibitors were found in bacteria and plants [9, 10, 12, 13, 19, 20–24, 26]. In some invertebrates, ChEs were significantly inhibited by eserine sulphate and BW284C51, but not by iso-OMPA [74, 75] however, the latter was not the case in the studies conducted by Varo et al. [7] and Diamantino et al. [6]. Limited inhibition by single inhibitors could also indicate a presence of more than one ChE-like enzyme in crude aqueous extracts which is in line with the multiple activity bands evident on the electrophoresis gels.

Cholinesterases have emerged from a common carboxylesterase superfamily [41], and are currently classified as members of the evolutionary conservative α/β-fold hydrolase family of proteins [78–80], which are comprised of thousands of catalytic and non-catalytic members. Proteins of the α/β-fold hydrolase family can act as hydrolases, lipases, transferases, hormone precursors or transporters, adhesion proteins, and chaperons of other proteins routes [80, 81]. Some fungal lipases show remarkable similarity to *T*. *californica* AChE, including the presence of a catalytic triad Ser-His-Glu [62, 63]. Considering this, and based on the findings by Sánchez et al. [13], who showed that ChEs from *Pseudomonas aeruginosa* can hydrolyse *p*-nitrophenyl butyrate and *p*-nitrophenyl propionate, an artificial substrates for lipases, we also checked whether our aqueous mushroom extracts exert lipase activity. Lipase activity was detected in nearly all the analysed mushroom samples, however, it did not correlate with the ChE-like activity, which was restricted to only 10 extracts.

It is known that mushrooms contain a number of substances with various biological activities including immunity-stimulating, antitumor, antimicrobial, antioxidant, anti-diabetic, anti-hyperlipidemic, anti-hypercholesterolemic, hepatoprotective, and anti-inflammatory effects. Mushrooms were also found to synthesize AChE inhibitors, and this class of compounds is used clinically to counteract various pathologies [60,82]. For this reason, we investigated whether mushrooms extracts can inhibit AChE from *D*. *melanogaster*, and we expected that the presence of inhibitors would be negatively correlated to the intrinsic ChE-like activity of extracts. Inhibitory activity against *D*. *melanogaster* AChE was confirmed in 10 extracts (Fig 5, Table 1). Surprisingly, this activity was detected both in extracts exhibiting ChE-like activity, e.g. in *Cortinarius purpurascens* (17), *Clitocybe phaeophthalma* (31), and *Hygrophorus aurantiaca* (37), and in those lacking the ChE-like activity, e.g. in *Amanita excelsa* (29) and *Entoloma rhodopolium* (1). It is nevertheless possible that other extracts also contain ChE inhibitors that display narrow specificity against other ChE types not tested in this work (e.g. not from *D*. *melanogaster*). These AChE-inhibitory properties were not restricted to any family of mushrooms but rather present across all included families. The highest inhibition was recorded with *Entoloma rhodopolium*, which is known to harbour muscarine, an agonist of the muscarine receptors but with no previously recorded (A)ChE-inhibitory action [83]. Unlike using cold aqueous extraction as performed in our study, AChE inhibitors from other mushrooms belonging to Basidiomycota were identified using organic or hot water extracts from *Cortinarius infractus* (Cortinariaceae) [84], *Funalia trogii* (Polyporaceae) and *Ganoderma lucidum* (Ganodermataceae) [85, 86], *P*. *ostreatus* [86, 87], *Pleurotus pulmonarius* [59], *Polyporus sulphureus* and *Trametes versicolor* (both Polyporales) [88].

In conclusion, this study revealed that some Basidiomycota aqueous extracts possess ACh, BCh and PCh hydrolysing activity with the highest activity being recorded for ACh. Therefore, we conclude that Basidiomycota crude extracts contain ChE-like enzymes. None of the extracts was characterised as harbouring vertebrate-like AChE, since (i) ChE activities were not inhibited at high substrate concentrations, (ii) vertebrate AChE inhibitors neostigmine, an analogue of eserine, and BW284c51 had no inhibitory action on mushroom ChE-like activity, and (iii) some extracts were inhibited by the vertebrate BChE inhibitor iso-OMPA. Mushroom ChE-like enzymes therefore resemble neither vertebrate AChE nor BChE, but hold intermediary characteristics of both enzymes, as has commonly been reported for invertebrates. The lack of functional similarity of these enzymes to vertebrate ChEs was corroborated by phylogenetic analysis of putative ChE proteins in the fungal kingdom that revealed no clear orthologs of ChE enzymes in fungi. These results, together with the in-gel ChE-like activity detection suggesting different molecular masses, isoelectric points and/or oligomeric forms of mushrooms, imply that a great species-specific variability of ChE-like activities in mushrooms exists. Limited inhibition by single inhibitors and multiple activity bands using in-gel detection indicate a potential presence of more than one ChE-like enzyme in crude extracts. At this stage, we cannot completely exclude the possibility that the observed ability of mushroom extracts to hydrolyse choline esters derives from esterases different from ChEs. However, this study can serve as a platform for further research on ChE distribution, diversification and physiological roles in fungi. Purification and sequence analysis of mushroom extracts showing ChE-like activity that will help in associating the observed carboxylesterase activity in mushrooms to the presence of ChEs will be the main goal of our further studies.

## Supporting information

S1 Fig In-gel cholinesterase-like activity with acetylthiocholine chloride (ACh) as a substrate measured in aqueous extracts from selected species of Basidiomycota. Native polyacrylamide gel run at pH 4 and stained in maleate buffer, pH 6 with 5 mM ACh at room temperature for 2h. Electric eel AChE was used as control (C). Weak activity bands are marked with black arrows. The absence of the eeAChE signal in gel can be due to the lower stability of eeAChE in acidic pH, as already reported for other vertebrate AChEs (Akman et al. 2009; Ahmed et al. 2012), or to its limited migration into the polyacrylamide gel. IDs represent the following species: 15-*Lepiota brunneoincarnata*; 13-*Echinoderma asperum*; 32-*Echinoderma echinaceum*, 31-*Clitocybe phaeophthalma*; 17-*Cortinarius purpurescens*; 18-*Cortinarius variicolor*; 28-*Caloboletus calopus*; 19-*Gomphidius glutinosus*; 37-*Hygrophoropsis aurantiaca*.
(DOCX)

S2 Fig SDS-PAGE analysis of the proteins exhibiting cholinesterase-like activity with acetylthiocholine chloride (ACh). It was measured in crude extracts from *Echinoderma echinaceum* (32) *and Hygrophoropsis aurantiaca* (37). After Native PAGE of the crude extract the ChE activity band was excised, eluted overnight by diffusion from gel pieces and analysed by SDS-PAGE and silver staining. M, molecular mass marker.
(DOCX)

S3 Fig Gene tree of the family of carboxylesterases. The known animal cholinesterase proteins are indicated in red, human non-cholinesterase homologs in blue and fungal homologs in black. The proteins were identified based on the presence of a conserved Pfam domain PF00135.
(DOCX)

S1 Table Predicted putative ChEs from 12 basidiomycetes, 5 ascomycetes and 3 early diverging fungi. (XLSX)

S2 Table Known animal AChEs. (XLSX)
