# Supplementary material for: First evidence of cholinesterase-like activity in Basidiomycota
Source: PLoS One. 2019 Apr 30;14(4):e0216077. doi: 10.1371/journal.pone.0216077 (PMC6490906; doi:10.1371/journal.pone.0216077)
Supplement: S1 Fig — Native polyacrylamide gel run at pH 4 and stained in maleate buffer, pH 6 with 5 mM ACh at room temperature for 2h. Electric eel AChE was used as control (C). Weak activity bands are marked with black arrows. The absence of the eeAChE signal in gel can be due to the lower stability of eeAChE in acidic pH, as already reported for other vertebrate AChEs (Akman et al. 2009; Ahmed et al. 2012), or to its limited migration into the polyacrylamide gel. IDs represent the following species: 15-Lepiota brunneoincarnata; 13-Echinoderma asperum; 32-Echinoderma echinaceum, 31-Clitocybe phaeophthalma; 17-Cortinarius purpurescens; 18-Cortinarius variicolor; 28-Caloboletus calopus; 19-Gomphidius glutinosus; 37-Hygrophoropsis aurantiaca. (DOCX) [file pone.0216077.s001.docx]

**
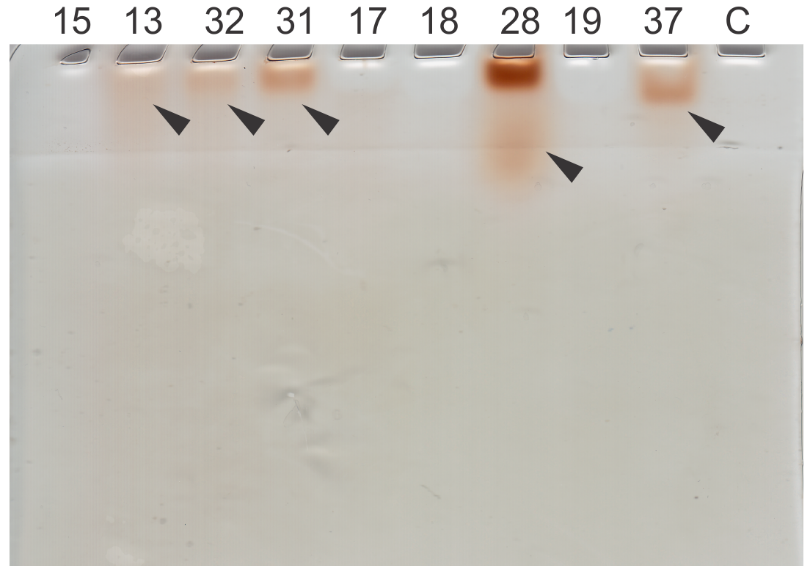
**

**S1 Figure**. **In-gel cholinesterase-like activity with acetylthiocholine chloride (ACh) as a substrate measured in aqueous extracts from selected species of Basidiomycota.** Native polyacrylamide gel run at pH 4 and stained in maleate buffer, pH 6 with 5 mM ACh at room temperature for 2h. Electric eel AChE was used as control (C). Weak activity bands are marked with black arrows. The absence of the eeAChE signal in gel can be due to the lower stability of eeAChE in acidic pH, as already reported for other vertebrate AChEs (Akman et al. 2009; Ahmed et al. 2012), or to its limited migration into the polyacrylamide gel. IDs represent the following species: 15-*Lepiota brunneoincarnata*; 13-*Echinoderma asperum*; 32-*Echinoderma echinaceum*, 31-*Clitocybe phaeophthalma*; 17-*Cortinarius purpurescens*; 18-*Cortinarius variicolor*; 28-*Caloboletus calopus*; 19-*Gomphidius glutinosus*; 37-*Hygrophoropsis aurantiaca*.

**References:**

Akman E, Turkoglu V, Celik I. Purification and characterization of Van lake fish (*Chalcalburnus tarichii* P.1811) liver and brain acethylcholinesterase. Hacettepe J. Biol. & Chem., 2009, 37 (4): 331-336.

Ahmed M, Latif N, Khan RA, Ahmad A, Rocha JBT, Mazzanti CM, Bagatini MD, Morsch VM, Schetinger MRC. Enzymatic and biochemical characterization of *Bungarus sindanus* snake venom acetylcholinesterase. J Venom Anim Toxins incl Trop Dis, 2012, 18: 236-243.
