# Supplementary material for: First evidence of cholinesterase-like activity in Basidiomycota
Source: PLoS One. 2019 Apr 30;14(4):e0216077. doi: 10.1371/journal.pone.0216077 (PMC6490906; doi:10.1371/journal.pone.0216077)
Supplement: S2 Fig — It was measured in crude extracts from Echinoderma echinaceum (32) and Hygrophoropsis aurantiaca (37). After Native PAGE of the crude extract the ChE activity band was excised, eluted overnight by diffusion from gel pieces and analysed by SDS-PAGE and silver staining. M, molecular mass marker. (DOCX) [file pone.0216077.s002.docx]

**
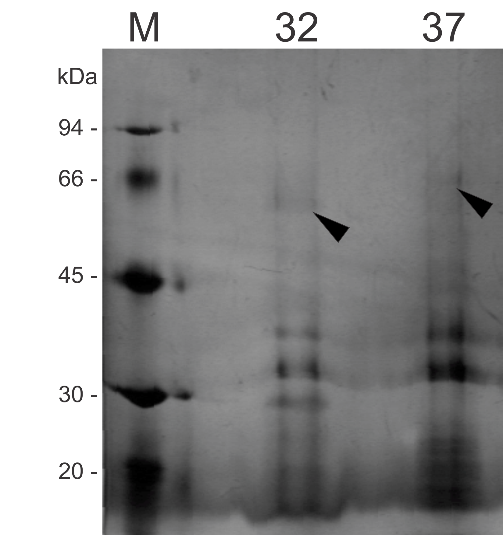
**

**S2 Figure.** **SDS-PAGE analysis of the proteins exhibiting cholinesterase-like activity** with acetylthiocholine chloride (ACh) as a substrate measured in crude extracts from *Echinoderma echinaceum* (32) *and Hygrophoropsis aurantiaca* (37). After Native PAGE of the crude extract the ChE activity band was excised, eluted overnight by diffusion from gel pieces and analysed by SDS-PAGE and silver staining. M, molecular mass marker.
