# Supplementary material for: First evidence of cholinesterase-like activity in Basidiomycota
Source: PLoS One. 2019 Apr 30;14(4):e0216077. doi: 10.1371/journal.pone.0216077 (PMC6490906; doi:10.1371/journal.pone.0216077)
Supplement: S3 Fig — The known animal cholinesterase proteins are indicated in red, human non-cholinesterase homologs in blue and fungal homologs in black. The proteins were identified based on the presence of a conserved Pfam domain PF00135. (DOCX) [file pone.0216077.s003.docx]

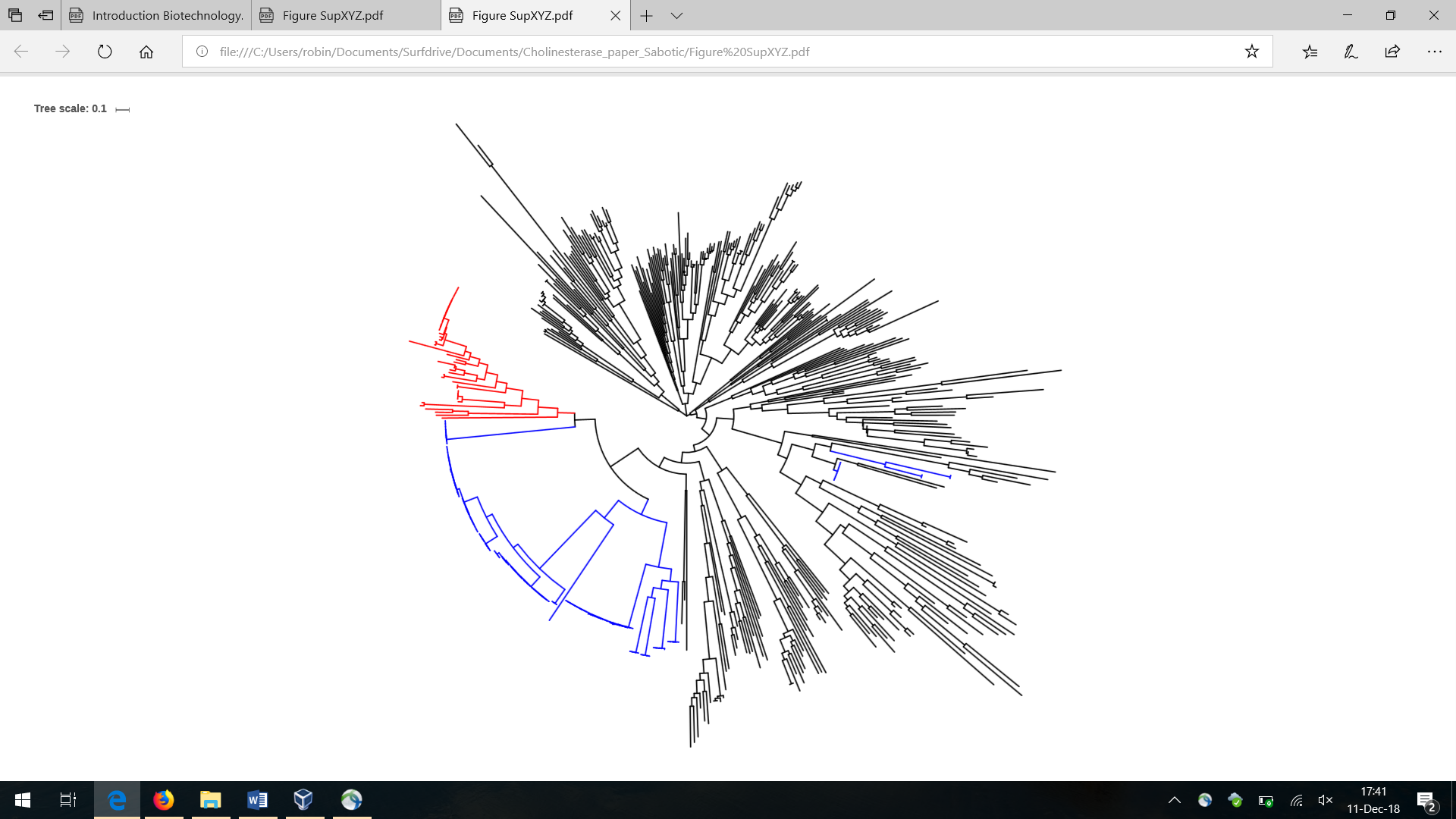


**S3 Figure.** **Gene tree of the family of carboxylesterases.** The known animal cholinesterase proteins are indicated in red, human non-cholinesterase homologs in blue, and fungal homologs in black. The proteins were identified based on the presence of a conserved Pfam domain PF00135.
